# The Impact of the Association Between Nonalcoholic Fatty Liver Disease and Intrahepatic Cholestasis of Pregnancy on Maternal and Fetal Outcomes

**DOI:** 10.7759/cureus.46035

**Published:** 2023-09-26

**Authors:** AK Koushik, Srilakshmidevi Kanumilli, Veera Abhinav Chinta, Yash R Shah, P Ganesh, Shanmughanathan Subramanyam

**Affiliations:** 1 Department of Gastroenterology, Sri Ramachandra Institute of Higher Education and Research, Chennai, IND; 2 Department of Internal Medicine, GSL Medical College, Rajahmundry, IND; 3 Department of Internal Medicine, Wayne State University/Trinity Health Oakland, Pontiac, USA

**Keywords:** fatty liver, outcomes, pregnancy, nonalcoholic fatty liver disease, intrahepatic cholestasis of pregnancy

## Abstract

Backgroundː Intrahepatic cholestasis of pregnancy (ICP), a hepatic condition that causes severe itching in late pregnancy, is linked to nonalcoholic fatty liver disease (NAFLD) due to disrupted bile acid balance. It poses maternal risks such as preterm labor and gestational diabetes and fetal risks such as preterm birth and respiratory distress. The study examined NAFLD's impact on ICP in pregnant women, highlighting management and research implications.

Methodsː This retrospective study examined pregnant women (≥18 years) with ICP, assessing fatty liver with follow-up ultrasounds. Participants were divided into ICP only and ICP with fatty liver (FL) groups, excluding heavy alcohol users and incomplete data. Maternal age, medical history, and comorbidities were evaluated alongside abdominal ultrasounds to identify FL.

Resultsː In this study of 43 pregnant women, the mean maternal age was 27 years. Patients with ICP and FL had significantly higher bile acid levels than those with ICP alone. However, no significant differences were found between the two groups regarding the history of gestational diabetes mellitus (GDM), dyslipidemia, polycystic ovarian syndrome (PCOS), parity, and hypothyroidism. Among women with ICP and FL, 51.85% underwent lower segment cesarean section (LSCS), while 43.75% with ICP without FL underwent LSCS.

Conclusionsː ICP with FL did not show significant adverse effects on maternal and neonatal outcomes, including mode of delivery, gestational age, maternal complications, neonatal intensive care unit (NICU) admissions, and low birth weight (LBW) with asphyxia. However, additional research is required to fully comprehend the relationship between ICP, NAFLD, and their impact on pregnancy outcomes.

## Introduction

The liver holds the distinction of being the largest organ within the body and is responsible for the metabolism of nutrients and toxins [1]. During pregnancy, the liver is affected by physiological and hormonal changes. While pregnancy may not have any impact on the liver, it can impact preexisting hepatic disorders [2,3]. Liver diseases such as intrahepatic cholestasis of pregnancy (ICP), toxemias, and hemolysis, elevated liver enzymes, and low platelets (HELLP) syndrome can significantly impact the morbidity and mortality rates of both mother and fetus, ranging from 0% to 25% [3,4].

ICP, characterized by pruritus, elevated serum aminotransferases, and bile acids, typically occurs in the second or third trimester of pregnancy. The signs and symptoms usually resolve within 2-3 weeks after delivery [5]. The reported prevalence of ICP among women in India is approximately 1% [6]. The exact cause remains unknown, although genetic, hormonal, and external factors are believed to contribute to its development [7]. The characteristic features are pruritus affecting the palms, soles, extremities, and trunk but sparing the mucous membranes. The intensity of pruritus may vary throughout pregnancy, and it resolves after parturition [8,9]. It is the second most common cause of jaundice during pregnancy, following viral hepatitis, accounting for approximately 20% of cases [10]. Jaundice typically appears 2-4 weeks after the onset of pruritus, resolving within 1-4 weeks after childbirth [11]. Unlike viral hepatitis, patients with ICP experience typical features of obstructive jaundice such as pale stools and dark urine [3,12]. Although the precise pathophysiology of ICP remains incompletely comprehended, its symptoms and biochemical anomalies typically subside postpartum. This condition is linked to an increased susceptibility to adverse obstetric outcomes such as stillbirth, respiratory distress syndrome, meconium passage, and fetal asphyxiation. This discourse will encompass an exploration of the clinical presentation, diagnostic procedures, and management approaches concerning ICP [13].

Nonalcoholic fatty liver disease (NAFLD) is closely associated with metabolic syndrome and is considered its hepatic manifestation. This condition is strongly linked with obesity and diabetes mellitus [14]. The reported prevalence of adult NAFLD in India ranges from 6.7% to 55.1% [15]. Approximately one-third of individuals with asymptomatic elevations in liver enzymes are believed to have NAFLD as an underlying cause [16]. Increased insulin resistance is inherent throughout pregnancy, and maternal obesity during pregnancy further increases the risk of gestational diabetes [17]. Although obesity and gestational diabetes are known to have adverse effects on perinatal outcomes, it is unclear whether NAFLD has an independent association with more severe pregnancy-related problems. Dysregulation of bile acid homeostasis has been associated with NAFLD progression [18].

Ensuring psychological support for pregnant women with ICP is crucial, as it helps them better comprehend and manage the condition through informative education and regular monitoring, thereby alleviating fears and promoting a sense of security [13].

While previous studies have linked ICP to NAFLD, with increased rates of steatosis and liver imaging, the association between NAFLD and ICP has not been previously studied. Therefore, the objective of the current research was to study the correlation between NAFLD and ICP and evaluate outcomes and potential complications of pregnancy. Studying the correlation between NAFLD and ICP can provide valuable insights that can contribute to improved patient care and outcomes. It can aid in early identification, risk stratification, and counseling, as well as targeted management strategies and enhanced prenatal and post-pregnancy care for women affected by these liver conditions.

## Materials and methods

The retrospective observational study was conducted by the Department of Medical Gastroenterology at Sri Ramachandra Hospital, a tertiary care center in Chennai, between October 2018 and December 2020. The study aimed to examine pregnant women with ICP who delivered during the specified period. The sample size calculation was based on a 95% confidence interval with a 10% standard error, resulting in a required sample size of 97. However, due to limitations in obtaining this number from previous hospital records, we retrospectively enrolled all eligible patients between October 2018 and December 2020, resulting in a total of 43 participants. The inclusion and exclusion criteria used for the purpose of the study are described in Table 1. The patients who met the inclusion and exclusion criteria described in Table 1 were divided into two groups. Patients with ICP but without evidence of FL on the abdominal US were classified into ICP without FL, and patients with ICP and fatty liver detected on the abdominal US were classified into ICP with FL group.

**Table 1:** Inclusion and exclusion criteria SBA: serum bile acids, AST: aspartate aminotransferase, ALT: alanine aminotransferase, US: ultrasound

Inclusion criteria	Exclusion criteria
1. Pregnant women of age ≥ 18 years	1. Patients with alcohol intake ≥ 20 g/day
2. Diagnosis of intrahepatic cholestasis of pregnancy with or without fatty liver (SBA > 11 mol/L, ALT > 41 IU/L, AST > 35 IU/L); abdominal US was performed for evaluation of fatty liver	2. Patients with incomplete data

Data collection

The study collected records of pregnant women with ICP, both with and without FL, who delivered at Sri Ramachandra Hospital, Chennai, during the study period. The data collected for the purpose of the study were maternal age, bile acid levels, total and direct bilirubin levels, aspartate aminotransferase levels, alanine aminotransferase levels, alkaline phosphatase levels, presence or absence of comorbidities (gestational diabetes mellitus (GDM), hypothyroidism, dyslipidemia, and polycystic ovarian syndrome (PCOS)), mode of delivery, gestational age at delivery, maternal complications (antepartum hemorrhage (APH) and pregnancy-induced hypertension (PIH)), neonatal low birth weight, and low birth weight with asphyxia. Abdominal ultrasound findings were assessed for the presence of FL. All relevant information was documented on a predesigned and pretested proforma.

Statistical analysis

The collected data were entered and organized in a Microsoft Excel Worksheet (Microsoft Corporation, Redmond, WA, USA). Continuous variables were analyzed for normality using Statistical Package for the Social Sciences (SPSS) version 20.0 (IBM SPSS Statistics, Armonk, NY, USA). Normally distributed data were reported as mean±standard deviation (SD), while skewed data were reported as median and interquartile range (IQR). Categorical data were compared using the chi-square test and Fisher's exact, as appropriate. The Mann-Whitney U test or independent sample t-test was used for comparing continuous variables depending on the distribution of the data.

Ethical approval

The study obtained ethical clearance from the Institutional Human Ethics Committee at Sri Ramachandra Institute of Higher Education and Research in Chennai. Informed patient consent was waived as this is a retrospective study and there was no direct interaction with the patients. The IRB approval number for the study is CSP-MED/20/SEP/61/84.

## Results

A total of 43 patients were categorized into two groups based on their condition. The first group comprised pregnant women with ICP without FL, while the second group comprised pregnant women with ICP and FL, as confirmed by ultrasound (US) of the abdomen.

In Table 2, the clinical liver profile of the patients was examined. Upon analysis, it was found that all parameters, except maternal age, displayed a skewed distribution within the bilirubin levels and liver enzymes (p<0.05). This indicates that the observed distribution of these parameters significantly deviates from a normal distribution, suggesting nonsymmetrical or skewed data patterns.

**Table 2:** Clinical profile of patients IQR: interquartile range, SD: standard deviation

Parameters and normal values	Mean (n=100)		Median	IQR	Range		p-value
	Mean	SD			Minimum	Maximum	
Maternal age (years)	27.58	3.88	27	5	19	38	0.261
Bile acids (2-5 mg/dL)	36.77	29.62	30	19.70	12.40	177.80	<0.001
Total bilirubin (0.5-1.2 mg/dL)	0.87	1.37	0.61	0.43	0.30	9.43	<0.001
Direct bilirubin (0-0.3 mg/dL)	0.35	0.83	0.18	0.17	0.05	5.51	<0.001
Aspartate aminotransferase (0-35 IU/L)	56.16	65.24	32	27	13	271	<0.001
Alanine aminotransferase (0-41 IU/L)	63	97.22	27	34	5	360	<0.001
Alkaline phosphatase (45-129 IU/L)	197.70	88.89	185	104	48	465	0.047

Between the two groups, there was no statistically significant difference in terms of maternal age, as indicated by the p-value of 0.267. The median level of bile acids was significantly greater in the ICP with FL group than in the ICP without FL group (p=0.022). There were no statistically significant differences observed between the two groups for total bilirubin, direct bilirubin, aspartate aminotransferase (AST), alanine aminotransferase (ALT), and alkaline phosphatase (ALP), as indicated by the p-values ranging from 0.617 to 0.714 as shown in Table 3.

**Table 3:** Comparison of the clinical profile of patients with ICP without FL and ICP with FL ICP: intrahepatic cholestasis of pregnancy, FL: fatty liver, IQR: interquartile range

Parameters and normal values	Liver disease				p-value
	ICP without FL (n=22)		ICP with FL (n=21)		
	Median	IQR	Median	IQR	
Maternal age (years)	25.5	4.75	28	5.5	0.267
Bile acids (2-5 mg/dL)	26.15	15.33	37	31.75	0.022
Total bilirubin (0.5-1.2 mg/dL)	0.64	0.27	0.55	0.65	0.696
Direct bilirubin (0-0.3 mg/dL)	0.18	0.12	0.17	0.41	0.617
Aspartate aminotransferase (0-35 IU/L)	30.5	14.5	36	82.5	0.705
Alanine aminotransferase (0-41 IU/L)	27	14.25	44	92.5	0.696
Alkaline phosphatase (45-129 IU/L)	170.5	120	187	118	0.714

In this study (Table 4), it was observed that 55.81% of the women had primiparity (first-time pregnancy), while 44.19% had multiparity (multiple pregnancies). Among women with primiparity, 54.17% had ICP with FL. However, no significant association was found between parity and the presence of ICP with FL (p=0.543).

**Table 4:** Distribution of patients according to gravida and history of GDM and its association with NAFLD ICP: intrahepatic cholestasis of pregnancy, FL: fatty liver, GDM: gestational diabetes mellitus, NAFLD: nonalcoholic fatty liver disease

	Liver disease				Total		p-value
	ICP without FL (n=22)		ICP with FL (n=21)				
	Number	%	Number	%	Number	%	
Gravida							
Primiparity	11	45.83	13	54.17	24	55.81	0.543
Multiparity	11	57.89	8	42.11	19	44.19	
Total	22	51.16	21	48.84	43	100	
Gestational diabetes mellitus							
Present	6	66.67	3	33.33	9	20.93	0.252
Absent	16	47.06	18	52.94	34	79.07	
Total	22	51.16	21	48.84	43	100	

Additionally, a history of gestational diabetes mellitus (GDM) was found in 20.93% of the women involved in the current research. Among women having a record of GDM, 33.33% had ICP with FL. However, no significant association was found between the history of GDM and the presence of ICP with FL (p=0.252).

Among the participants, 39.43% had a history of hypothyroidism, and within this group, 39.53% were found to have ICP with FL. However, statistical analysis did not reveal a notable correlation between a history of hypothyroidism and the occurrence of ICP with FL (p=0.124).

Furthermore, 23.26% of the women had a history of dyslipidemia, and among them, 70% were diagnosed with ICP with FL. Nevertheless, no significant association was found between a history of dyslipidemia and the presence of ICP with FL (p=0.121). In terms of polycystic ovarian syndrome (PCOS), only 2.33% of the women had a history of PCOS, and none of them were identified as having ICP with FL. Similar to the previous conditions, no significant association was found between a history of PCOS and the occurrence of ICP with FL (p=0.252) as shown in Table 5.

**Table 5:** Association of history of hypothyroidism, dyslipidemia, and PCOS with NAFLD ICP: intrahepatic cholestasis of pregnancy, FL: fatty liver, PCOS: polycystic ovarian syndrome, NAFLD: nonalcoholic fatty liver disease

	ICP without FL (n=22)		ICP with FL (n=21)		Total		p-value
	Number	%	Number	%	Number	%	
Hypothyroidism							
Present	6	35.29	11	64.71	17	39.53	0.124
Absent	16	61.54	10	38.46	26	60.47	
Total	22	51.16	21	48.84	43	100	
Dyslipidemia							
Present	3	30	7	70	10	23.26	0.121
Absent	19	57.58	14	42.42	33	76.74	
Total	22	51.16	21	48.84	43	100	
PCOS							
Present	1	100	0	0	1	2.33	0.512
Absent	21	50	21	50	42	97.67	
Total	22	51.16	21	48.84	43	100	

Table 6 depicts the mode of delivery that was evaluated among the participants. Among women with ICP and FL, 51.85% underwent emergency lower segment cesarean section (LSCS), whereas among women with ICP only, 43.75% underwent emergency LSCS. However, statistical analysis did not reveal a significant difference between the two groups in terms of the mode of delivery (p=0.607).

**Table 6:** Association of mode of delivery and gestational age with NAFLD ICP: intrahepatic cholestasis of pregnancy, FL: fatty liver, NAFLD: nonalcoholic fatty liver disease, LSCS: lower segment cesarean section

	ICP only (n=22)		ICP with FL (n=21)		Total		p-value
	Number	%	Number	%	Number	%	
Mode of delivery							
Normal vaginal delivery	9	56.25	7	43.75	16	37.21	0.607
Emergency LSCS	13	48.15	14	51.85	27	62.79	
Total	22	51.16	21	48.84	43	100	
Gestational age at delivery							
Full term	15	57.69	11	42.31	26	60.47	0.358
Preterm	7	41.18	10	58.82	17	39.53	
Total	22	51.16	21	48.84	43	100	

The study also examined gestational age at delivery. It was found that 60.47% of the women delivered full-term neonates, while 58.82% of women with ICP and FL delivered preterm neonates. However, no significant association was found between gestational age at delivery and the presence of ICP with FL (p=0.358).

The aim of this study was to examine maternal complications, neonatal intensive care unit (NICU) admission, and the incidence of low birth weight (LBW) and LBW with birth asphyxia in neonates born to patients with ICP and FL. Among the study participants (Table 7), 18.60% experienced maternal complications, while 62.50% of those with ICP and FL had such complications. However, the statistical analysis did not reveal a statistically significant association between ICP with FL and maternal complications. These results suggest that the presence of ICP with FL does not significantly contribute to an increased risk of maternal complications. 

**Table 7:** Association of maternal complications, NICU admission, and neonatal complications with NAFLD ICP: intrahepatic cholestasis of pregnancy, FL: fatty liver, PIH: pregnancy-induced hypertension, APH: antepartum hemorrhage, NICU: neonatal intensive care unit, NAFLD: nonalcoholic fatty liver disease

	Liver disease				Total		p-value
	ICP without FL (n=22)		ICP with FL (n=21)				
	Number	%	Number	%	Number	%	
Maternal complications (PIH and APH)							
Present	3	37.50	5	62.50	8	18.60	0.391
Absent	19	54.29	16	45.71	35	81.40	
Total	22	51.16	21	48.84	43	100	
NICU admission							
Required	5	31.25	11	68.75	16	37.21	0.062
Not required	17	62.96	10	37.04	27	62.79	
Total	22	51.16	21	48.84	43	100	
Neonatal complications							
Low birth weight	7	46.67	8	53.33	15	34.88	0.773
Low birth weight with birth asphyxia	3	42.86	4	57.14	7	16.28	
Absent	12	57.14	9	42.86	21	48.84	
Total	22	51.16	21	48.84	43	100	

Regarding neonatal outcomes, 37.21% of all neonates required admission to NICU, with 68.75% of neonates born to women with ICP and FL needing admission. However, the analysis did not find a significant association between ICP with FL and NICU admission. This implies that the presence of ICP with FL in pregnant patients does not significantly impact the likelihood of NICU admission. Furthermore, no significant associations were observed between ICP with FL and the occurrence of LBW or LBW with birth asphyxia. These findings suggest that ICP with FL does not significantly contribute to adverse maternal or neonatal outcomes related to LBW or LBW with birth asphyxia.

## Discussion

ICP is a common hepatic condition encountered during pregnancy. Diagnosis is typically made based on the clinical presentation, which includes pruritus, impairment in liver function tests, and frequently elevated serum levels of total bile acids in the serum. In Indian women, the reported incidence of ICP is approximately 1%. However, in our study, we observed a relatively higher incidence of 2.4%. The mean age of the women in our study was 26.3 years, which is comparable to some studies, although other studies have reported a relatively higher mean age. Additionally, we found that 18% of women had a family history of ICP, and recurrence of ICP was noted in 50% of them. This indicates a potential genetic predisposition to the condition. The majority of women in our study (96%) presented after completing 30 weeks of pregnancy, which is consistent with findings from other studies [6]. Furthermore, pregnancy-related NAFLD poses increased risks for both maternal and fetal health, such as hypertensive complications, postpartum bleeding, and preterm birth [19].

The current study included 43 pregnant women, with a median maternal age of 27 years. The participants were divided into two groups: ICP without FL and ICP with FL. The study findings revealed that patients with ICP and FL had significantly higher median bile acid levels (p=0.022) compared to those without FL. However, other liver profile parameters such as total bilirubin, direct bilirubin, and liver enzymes such as AST, ALT, and ALP were statistically similar in both cohorts. These results suggest that the presence of FL is more likely to occur in pregnant women with ICP who also have increased bile acid levels. These findings relate to a previous study by Monrose et al. (2019), which reported a connection between impaired bile acid metabolism and both liver disease and pregnancy-related cholestasis [20]. The study by Monrose et al. (2019) observed higher ALT levels exceeding two times the upper limits of normal, suggesting that it will be helpful in screening pregnant patients who are diagnosed with ICP and have a concurrent elevation of ALT to be screened for NAFLD, which was not evident in the current investigation [20].

In this study, it was found that slightly over half of the women (55.81%) were categorized as primipara. Among women with ICP and FL, 54.17% were primiparous, while the percentage for women with ICP was only 45.83%. However, the statistical analysis indicated that this difference was not statistically significant (p=0.543), suggesting no association between parity and ICP with FL. Furthermore, 20.93% of the women had a history of GDM, and among them, 33.33% had ICP with FL. However, the analysis did not find a significant association between the history of GDM and the presence of ICP with FL (p=0.252). These results were concurrent with previous research conducted by Monrose et al. in 2019 [20]. This study explores the association between NAFLD and ICP and their impact on maternal and fetal outcomes. This unique research sheds light on the potential complications and challenges faced by women who have both conditions during pregnancy. By enhancing our understanding of liver disorders in pregnancy, this study contributes to improving clinical management and interventions for affected women. Further research is needed to deepen our knowledge of this complex relationship.

In the current study, a significant proportion of the women had various comorbidities, including gestational diabetes mellitus (GDM) (20.93%), hypothyroidism (39.53%), dyslipidemia (23.26%), and polycystic ovarian syndrome (PCOS) (2.33%). However, the statistical analysis did not reveal any significant correlation between the presence of ICP with FL and these comorbidities (p=0.512). These findings align with a study conducted by Kushner et al. in 2023, where no correlation was found between a history of cholestasis during pregnancy, gestational hypertension, preterm delivery, hemolysis, elevated liver enzymes, low platelet count, acute fatty liver during pregnancy, or other reported pregnancy complications [19].

Interestingly, these findings differ from those reported by Koralegedara et al., who demonstrated a significant association between nonalcoholic fatty liver disease (NAFLD) and an increased risk of GDM and early pregnancy miscarriages. The presence of NAFLD was found to contribute to a state of insulin resistance, leading to a higher likelihood of developing hyperglycemia during pregnancy [21]. It is worth noting that the results of the present study did not align with these previous findings.

These observations emphasize the complex nature of the relationships between ICP with FL and comorbidities such as GDM, hypothyroidism, dyslipidemia, and PCOS. Although no significant associations were found in the current study, further research is needed to explore the potential interactions, underlying mechanisms, and implications of these comorbidities in the context of ICP with FL. Continued investigation will provide a more comprehensive understanding of the interplay between liver disorders and other maternal conditions, allowing for improved management strategies and interventions in the future.

In this study, it was observed that the majority of women (62.79%) who underwent emergency lower segment cesarean section (LSCS) had favorable maternal outcomes. Although women with ICP and FL had slightly higher rates of emergency LSCS (51.85%) compared to women with ICP alone (48.15%), the difference was not statistically significant (p=0.607). These findings indicate that there was no correlation between the mode of delivery and the presence of ICP with FL.

The overall rate of LSCS deliveries among pregnant women with ICP in the present study was similar to the findings reported in other investigations that focused solely on pregnant women with ICP but without FL. For instance, Alakananda et al. (2016) [22], Mahajan et al. (2017) [23], and Nisha et al. (2019) [24] reported LSCS rates of 44.5%, 46.81%, and 38.67%, respectively, in their studies. It is important to note that these studies exclusively included pregnant women with ICP without FL.

These observations suggest that the presence of FL does not significantly impact the mode of delivery or maternal outcomes in pregnant women with ICP. The results of this study align with previous research that also did not find a statistically significant difference in the mode of delivery between women with ICP and those with ICP and FL.

The risk of preterm birth had been linked to ICP ranging from 19% to 60%. The majority of the women in the current study (60.47%) delivered full-term newborns. However, compared with 41.18% of the women with ICP without FL, nearly two-thirds (58.82%) of the women with ICP with FL delivered premature neonates. However, the difference was statistically insignificant (p=0.358). These findings imply that there was no correlation between gestational age at delivery and ICP with FL (p=0.358). Of the neonates, 40.32% were reported to be born prematurely by Parihar and Singh (2019) [3]. Mahajan et al. (2017) reported a rate of preterm delivery that was lower (10.67%) than that of the current study [23]. Within this study, 18.60% of the women experienced maternal complications, and 62.50% of the women with complications had ICP with FL. However, no significant association was found between maternal complications and ICP with FL (p=0.391). Other studies focusing on pregnant women with ICP have reported a range of complications. Moreover, in this study, over one-third of the neonates (37.21%) required admission to the NICU. Among the neonates delivered by women with ICP and FL, the majority (68.75%) necessitated NICU care, whereas a smaller proportion of neonates delivered by women with ICP alone (31.25%) required such care. The statistical analysis did not reveal a significant association between the requirement for NICU admission and ICP with FL (p=0.062). However, another study reported lower rates of NICU admission, which were very low compared with studies of Mahajan et al. (2017) [23] and Padmaja et al. (2010) [25].

The underlying mechanisms leading to fetal complications in the context of ICP with FL remain insufficiently understood. In our current study, a significant proportion of neonates (34.88%) were classified as having low birth weight (LBW), indicating a potential impact on fetal growth. Furthermore, a subset of neonates (16.28%) experienced both LBW and birth asphyxia, highlighting the presence of additional challenges. Among neonates with LBW, slightly over half (53.33%) were born to women who had ICP with FL, suggesting a potential association between these conditions. Similarly, a majority of neonates (57.14%) who experienced both LBW and birth asphyxia were born to women with ICP and FL. Although the statistical analysis did not demonstrate significant associations between ICP with FL and LBW or LBW with birth asphyxia (p=0.773), the observed trends suggest a potential relationship that warrants further investigation. The occurrence of LBW in our study is consistent with findings from a study conducted by Parihar and Singh in 2019, where 45.16% of newborns were classified as LBW [3]. These parallel observations suggest that LBW is a common outcome in pregnancies complicated by ICP with FL, further highlighting the need to understand the underlying mechanisms and identify potential interventions to mitigate these risks.

The results of this study will play a crucial role in the management of patients who are diagnosed with ICP and are diagnosed with concomitant NAFLD during pregnancy. As it is evident that there are no significant adverse pregnancy or neonatal outcomes in patients with coexisting ICP and NAFLD, it will be beneficial during prenatal care and counseling of the patients. The symptoms can be effectively managed by addressing the physical discomfort caused by itching. Skillfully managing these symptoms can indirectly lead to an enhancement in their psychological well-being [26].

Prenatal counseling assumes a critical role for pregnant women serving as a platform to delve into their emotional state, apprehensions, and concerns linked to the condition. Counselors can offer tailored coping strategies, stress reduction techniques, and emotional bolstering [27]. Providing a secure environment wherein pregnant women can freely express their emotions, apprehensions, and worries can offer immense advantages. This support can be extended by healthcare practitioners, partners, family members, or even support groups. Enlisting the expertise of professional mental health services, such as counseling or therapy, can equip pregnant women with effective tools to cope with anxiety, depression, and other emotional trials that may stem from ICP [28]. Counseling the patients that concomitant ICP and NAFLD will not impact pregnancy or neonatal outcomes can play a crucial role in managing peripartum depression, anxiety, or mental health disorders. Involving partners and family members in the process can foster a robust support system. Encouraging open dialogue and empathy within the family can alleviate stress. This form of counseling takes on paramount importance within the context of ICP, as it addresses not only the physical health aspects but also the psychological and emotional well-being of expectant mothers. It recognizes the potential psychological hurdles linked to ICP and equips individuals with effective mechanisms to handle them. Ultimately, prenatal counseling contributes to a more positive pregnancy journey, enhances mental well-being, and augments overall outcomes for both the mother and the baby.

It is important to consider the limitations of the study, such as the relatively small sample size from a single center and retrospective study design. Additionally, other metabolic risk factors such as obesity, hypertension, and particular forms of dyslipidemia were not considered within the scope of the study; thus, they were not taken into account as a part of the analysis. Further research with larger sample sizes and comprehensive investigations is warranted to confirm these findings and explore potential associations between ICP with FL and other relevant outcomes. 

## Conclusions

Intrahepatic cholestasis of pregnancy (ICP) is the leading cause of hepatic impairment in pregnancy. ICP seems to be a significant risk factor for FL development. Bile acid levels were noted to be significantly higher in patients in the ICP with FL group as compared to patients in the ICP without FL group. The additional maternal risk factors tested in patients in the ICP with FL group did not show any significant impact on the maternal and fetal outcomes as compared to the patients in the ICP without FL group. However, it is worth noting that a substantial proportion of neonates born to women with ICP and FL experienced LBW and birth asphyxia, although the statistical analysis did not reveal significant associations. Overall, our study contributes to the growing body of knowledge on liver disorders in pregnancy, specifically focusing on ICP with FL. It highlights the need for early detection, appropriate management, and interventions to optimize maternal and fetal health outcomes.
